# The 3.1-Angstrom Cryo-electron Microscopy Structure of the Porcine Epidemic Diarrhea Virus Spike Protein in the Prefusion Conformation

**DOI:** 10.1128/JVI.00923-19

**Published:** 2019-11-13

**Authors:** Daniel Wrapp, Jason S. McLellan

**Affiliations:** a Department of Molecular Biosciences, The University of Texas at Austin, Austin, Texas, USA; Loyola University Chicago

**Keywords:** PEDV, S1-CTD, alphacoronavirus spike, cryo-EM

## Abstract

Porcine epidemic diarrhea virus (PEDV) is an alphacoronavirus that has a significant agricultural and economic impact due to the high mortality rate associated with infection of neonatal piglets. Like other coronaviruses, PEDV makes use of a large, trimeric spike (S) glycoprotein to mediate membrane fusion and gain entry into host cells. Despite the importance of the spike protein in viral entry and host immune responses, high-resolution structural information concerning this large macromolecular machine has been difficult to obtain. Here, we report the cryo-electron microscopy structure of the PEDV S protein in the prefusion conformation at a resolution of 3.1 Å. Our studies revealed that the sialic acid-binding domain at the N terminus of the S1 subunit has an orientation that is substantially different from that observed in the previously determined spike structure from human alphacoronavirus NL63. We also observed dissociated S1 subunit trimers wherein the putative receptor-binding domains exist in a conformation differing from that observed in the intact spike proteins, suggesting that the PEDV receptor-binding domain undergoes conformational rearrangements akin to those that have been described in the related betacoronaviruses. Collectively, these data provide new insights into the biological processes that mediate alphacoronavirus attachment, receptor engagement, and fusion triggering while also identifying a source of conformational heterogeneity that could be manipulated to improve PEDV vaccine antigens.

**IMPORTANCE** Coronavirus spike proteins are large, densely glycosylated macromolecular machines that mediate receptor binding and membrane fusion to facilitate entry into host cells. This report describes the atomic-resolution structure of the spike protein from porcine epidemic diarrhea virus, a pathogenic alphacoronavirus that causes severe agricultural damage. The structure reveals a novel position for the sialic acid-binding attachment domain in the intact spike. We also observed shed fusion-suppressive capping subunits that displayed the putative receptor-binding domain in an accessible conformation. These observations provide a basis for understanding the molecular mechanisms that drive the earliest stages of alphacoronavirus infection and will inform future efforts to rationally design vaccines.

## Abstract (precis)

Coronavirus spike proteins are large, densely glycosylated macromolecular machines that mediate receptor binding and membrane fusion to facilitate entry into host cells. This report describes the atomic-resolution structure of the spike protein from porcine epidemic diarrhea virus, a pathogenic alphacoronavirus that causes severe agricultural damage. The structure reveals a novel position for the sialic acid-binding attachment domain in the intact spike. We also observed shed fusion-suppressive capping subunits that displayed the putative receptor-binding domain in an accessible conformation. These observations provide a basis for understanding the molecular mechanisms that drive the earliest stages of alphacoronavirus infection and will inform future efforts to rationally design vaccines.

## INTRODUCTION

Porcine epidemic diarrhea virus (PEDV) is a highly virulent coronavirus that causes severe enteric disease in pigs (1). PEDV infection manifests itself as watery diarrhea and vomiting and leads to dangerous dehydration that is often fatal, particularly in neonatal piglets (2, 3). PEDV was first isolated in the United Kingdom in the early 1970s, where it was initially believed to be a new strain of transmissible gastroenteritis virus (TGEV) (4). PEDV outbreaks since 2010 in China, the United States, and Canada are estimated to have caused over $1 billion in agricultural damage, which has led to renewed interest in the development of antiviral therapeutics (5, 6). Reports of protection against PEDV reinfection and passive transfer of lactogenic immunity to suckling piglets suggest that vaccination would be an effective means of preventing future outbreaks, although there is currently no such approved vaccine in the United States or Europe (7). Recent efforts in Asia have produced both live attenuated and inactivated vaccines, but their efficacies have yet to be thoroughly evaluated (8).

PEDV is an alphacoronavirus with a 28-kbp positive-sense single-stranded RNA (ssRNA) genome that contains at least seven open reading frames (ORF) (9). ORF 2 encodes the fusion glycoprotein spike (PEDV S), which acts as both the major determinant of host cell tropism and the mediator of viral entry into host cells (10). PEDV S is a homotrimeric class I fusion protein that is processed into S1 and S2 subunits by trypsin-like host cell proteases (11). For most coronaviruses, the N-terminal domain (NTD) of the S1 subunit attaches to cellular carbohydrates and the C-terminal domain of S1 (S1-CTD) binds to a cellular protein receptor (12–16). Carbohydrate binding by the S1 N-terminal domain is thought to keep the virus in close proximity to the host cell surface, whereas engagement of specific protein receptors by the S1-CTD is thought to initiate a series of conformational changes in the spike that ultimately result in membrane fusion and delivery of the viral genome to the cytosol. The S2 subunit mediates the membrane fusion event and exhibits the characteristics that define class I fusion proteins. These include a hydrophobic fusion peptide that is directly C terminal to a protease cleavage site, as well as a series of α-helices in the prefusion conformation that rearrange to form a thermostable, elongated six-helix bundle in the postfusion conformation (17, 18).

One characteristic that distinguishes the majority of alphacoronavirus spikes from their homologs in other coronavirus genera is the presence of an additional domain (D∅) at the N terminus of S1 that arose from duplication of the NTD (19). PEDV S D∅ has been shown to preferentially bind to sialic acid, which is thought to heavily influence the host cell tropism of PEDV. This interaction has been characterized through both high-throughput glycan arrays and hemagglutination assays (20, 21). Although a precise, quantitative analysis of the affinity of this interaction has yet to be reported, it is generally thought to be a relatively low-affinity interaction that is facilitated by the avidity effect of having numerous copies of trimeric spikes present on the viral surface (22). Despite the importance of this carbohydrate-binding interaction in facilitating cellular attachment, the isolation of D∅-deletion variants *in vivo* and *in vitro* suggests that sialic acid binding is not strictly required for cell entry (23, 24). However, inoculation with D∅-deletion variants results in attenuated disease compared to strains that contain D∅ (25).

The engagement of a host cell protein receptor by the S1-CTD is thought to be strictly required in order for alphacoronavirus infection to occur. Although the crystal structures of the alphacoronavirus S1-CTDs from NL63, TGEV, and 229E in complex with their respective receptors have all been solved, the functional host cell receptor for PEDV remains unknown (15, 26–28). It has been suggested that PEDV makes use of porcine aminopeptidase N (pAPN) as a receptor; however, pAPN-knockout swine testis cells are still susceptible to PEDV infection, and this putative interaction has yet to be recapitulated with purified, recombinant components (12, 29). Regardless of the identity of its functional receptor, PEDV has been shown to infect and replicate in porcine, simian, and human cells, indicating that the virus likely makes use of receptors that share a high degree of homology among these species (21).

Recent structural characterizations of S proteins from the betacoronaviruses severe acute respiratory syndrome coronavirus (SARS-CoV) and MERS-CoV have revealed that the S1-CTDs from these spikes exist in a dynamic equilibrium between at least two distinct conformations. In one of these conformations, the S1-CTDs pack tightly against the S2 subunit, making the receptor-binding motifs inaccessible to host cell receptors and neutralizing antibodies. In the alternative conformation, the S1-CTD hinges away from the spike, such that it no longer associates with S2 and the receptor-binding motifs are no longer occluded (30–32). It has been proposed that sequential receptor binding events trap this transient, receptor-accessible conformation and gradually destabilize S, leading to dissociation of S1, refolding of S2, and membrane fusion (32, 33). However, in the only alphacoronavirus S structure reported to date, no such S1-CTD dynamics were reported and all three S1-CTDs were in a compact, receptor-inaccessible conformation (34). Although it is possible that the alphacoronaviruses make use of a triggering mechanism different from that used by the closely related betacoronaviruses, it seems more likely that this transient conformation simply has yet to be observed.

To learn more about the processes that mediate PEDV entry and infection, we produced the soluble ectodomain of the PEDV spike protein from the classical CV777 strain and solved the structure of this macromolecular machine to a resolution of 3.1 Å by cryo-electron microscopy (cryo-EM). The structure revealed a D∅ conformation that was distinct from that observed in the previously determined NL63 S structure. Additionally, particles of dissociated S1 rings with S1-CTDs in two different conformations were also observed, suggesting that alphacoronavirus spike-mediated fusion is initiated similarly to what has been proposed for betacoronaviruses.

## RESULTS

### Expression of PEDV CV777 S and cryo-EM sample preparation.

To structurally characterize the prefusion conformation of PEDV CV777 S, we expressed the soluble ectodomain, encompassing residues 1 to 1319. This construct was designed to be truncated just after heptad repeat 2 (HR2) such that the membrane-proximal external region (MPER), transmembrane domain (TM), and intravirion tail (IV) were excluded. To these residues we appended a C-terminal T4 fibritin trimerization motif, an 8× His tag, and a Twin-Strep-tag. Affinity purification from a 500-ml transfection volume yielded approximately 0.5 mg of protein, which was then further purified by size exclusion chromatography (SEC). SDS-PAGE revealed a single band between 160 and 220 kDa, corresponding to the molecular weight of a glycosylated monomer that showed no evidence of cleavage by endogenous proteases. The SEC chromatogram revealed a single, symmetrical peak with an elution volume corresponding to a molecular weight of roughly 600 kDa, suggesting a heavily glycosylated homotrimer. The purified protein was then used to prepare cryo-EM grids. Initially, CF-1.2/1.3 grids were used, but the PEDV S proteins were occluded from the thin ice and exhibited a tendency to cluster around the edge of the holes rather than being uniformly embedded throughout the vitreous ice layer. To mitigate these effects, CF-2/2 grids were prepared using optimized blotting conditions, which allowed collection of full fields of view that contained PEDV S proteins throughout.

### Structure determination of PEDV CV777 S.

**FIG 1:**
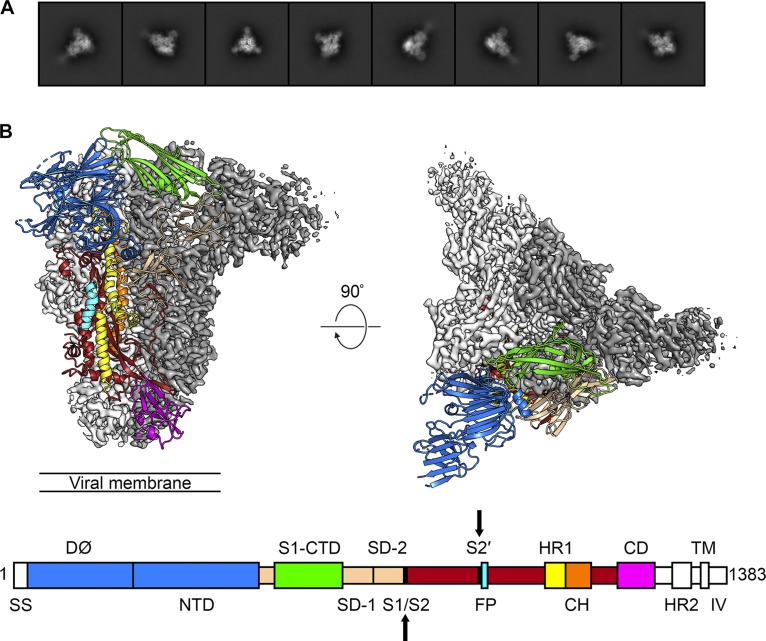
The 3.1-Å structure of PEDV S in the prefusion conformation. (A) 2D class averages determined for the PEDV S protein. (B) The densities for two protomers are shown, colored white and gray. The third protomer is shown as ribbons, colored by domain corresponding to the primary structure diagram. SS, signal sequence; S1/S2, S1/S2 protease cleavage site; S2′, S2′ protease cleavage site; FP, fusion peptide; HR1, heptad repeat 1; CH, central helix; CD, connector domain; HR2, heptad repeat 2; TM, transmembrane domain; IV, intravirion tail. Protease cleavage sites S1/S2 and S2′ are highlighted by arrows on the primary structure diagram.

Micrograph movies collected on a K2 Summit detector using a Titan Krios operating at 300 kV were first processed in *Warp* to perform motion correction, contrast transfer function (CTF) estimation, and BoxNet-based nontemplated particle picking (35). The 269,838 Warp-extracted particles were then imported into cryoSPARC v2 for further processing (36). Two-dimensional (2D) classification removed 122,440 “junk” particles, and the remaining 147,398 particles were used to generate three *ab initio* volumes without imposition of any symmetry restraints. These three volumes were then used as templates to sort all 147,398 particles by 3D classification. This more stringent classification method sorted 34,743 particles into two classes which did not resemble biological macromolecules. The remaining 112,655 particles were sorted into a third class in which secondary structural elements were clearly visible. This final set of 112,655 particles was used to perform nonuniform homogeneous 3D refinement. These data were initially processed without imposing symmetry, resulting in a 3.3-Å map with obvious C3 symmetry. After C3 symmetry was imposed, the resolution improved to 3.1 Å (Fig. 1; see also Fig. S2 in the supplemental material). This map was then sharpened using LocalDeBlur before being used for model building and refinement (37).

The PEDV CV777 S map has a length of ∼150 Å and a radius of ∼85 Å, measuring from the 3-fold axis of symmetry to the tip of D∅. Our final model contains 1,064 amino acids per monomer, spanning Thr43 to Tyr1242. The majority of the residues that are missing from our model are contained within D∅, where a relatively low (∼5-Å) local resolution prohibited unambiguous building of some regions. Similarly, the density began to fade after Tyr1242 and the flexible HR2 domain at the C terminus of S could not be resolved. Our model encompasses 21 of the 29 *N*-linked glycosylation sites on each protomer of the PEDV spike, with 16 of these contained within the S1 subunit and 5 contained within the S2 subunit. We observed density for and built corresponding glycan molecules at 18 of the 21 sites. For the majority of these sites, we built only a single *N*-acetylglucosamine, whereas larger glycan chains could be built at 5 sites (Fig. S4D). The PEDV S1 subunit is almost entirely composed of β-sheets, whereas the S2 subunit is made up of a series of discontinuous α-helices that are characteristic of a class I fusion protein in the prefusion conformation (Fig. S3A).

D∅ (Arg34 to Cys231) is the domain of PEDV S located farthest away from the central 3-fold axis of symmetry. Although we were unable to build roughly half of this domain, eight β-strands that are organized into two β-sheets are clearly observable. D∅ contains four *N*-linked glycosylation sites, two of which could be clearly resolved and one of which contained density corresponding to the glycan itself. The S1-NTD (Thr232 to Ser471) adopts a tertiary structure similar to that of D∅, forming 12 β-strands that are organized into two β-sheets. Overall, the density in this region allowed unambiguous model building, excluding a flexible loop at the apex of the trimer (Leu354 to Ala363) that could not be clearly resolved. The NTD is densely glycosylated, with seven *N*-linked glycosylation sites, all of which could be resolved and six of which contained corresponding glycan density. The density at Asn261 was particularly prominent and allowed us to build a branched, high-mannose glycan that was packed between the NTD and the α-helices of the S2 fusion machinery. Notably, similar elongated glycans have been observed playing the same structural role at Asn74 and Asn240 in the cryo-EM structures of porcine deltacoronavirus (PDCoV) S and NL63 S, respectively (34, 38).

**FIG 2:**
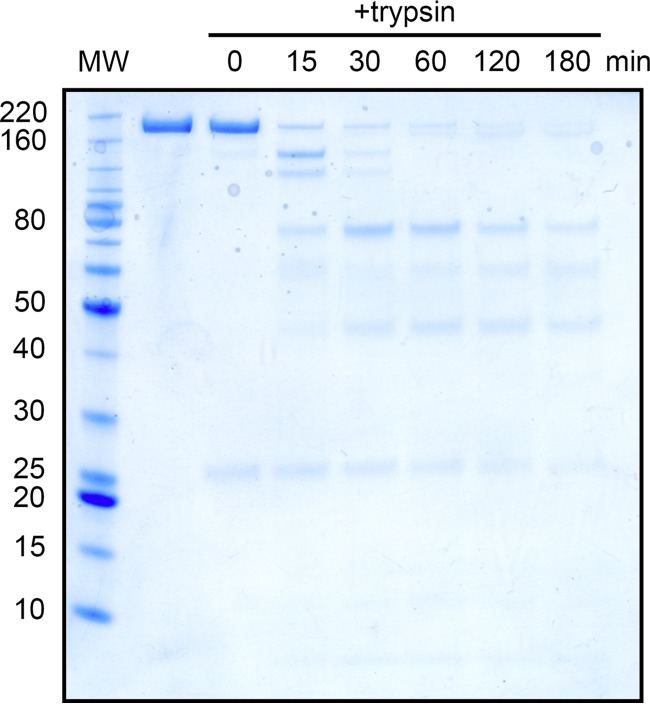
Trypsin digestion and SDS-PAGE analysis. A reducing SDS-PAGE gel is shown, with a ladder in the far-left (MW) lane. The molecular weights of the ladder are labeled in kilodaltons to the left of the gel. The lane immediately to the right of the ladder shows purified PEDV CV777 S in the absence of trypsin, and the lanes to the right of the uncleaved S sample lane were treated with trypsin for the number of minutes indicated at the top of the lane.

The S1-CTD (Phe504 to Asp637) is composed of 10 β-strands, a single α-helix, and 2 *N*-linked glycosylation sites, both of which could be observed in our map. Although a majority of the S1-CTD is organized into β-sheets, these secondary structural elements are thought to function as a scaffold for key aromatic residues based on the interactions between closely related alphacoronaviruses and their respective host cell receptors (15, 27). These aromatic residues are packed tightly against the NTD of the same protomer in the prefusion conformation, suggesting that the S1-CTD must undergo a conformational rearrangement in order to productively engage a host cell receptor. Subdomain 1 (SD-1) and subdomain 2 (SD-2) (Gln472 to Ser503 and Val638 to Tyr761, respectively) are composed of 14 β-strands and 5 *N*-linked glycosylation sites, 4 of which had observable glycan density. The putative S1/S2 cleavage site, between Lys755 and Ser756, is located in a small loop between the final two β-strands of SD-2 (Fig. S4A and B) (11). This cleavage site is positioned at the side of PEDV S, roughly equidistant between the viral membrane and the apex of the spike. Although this site is readily accessible to host trypsin-like proteases and can be cleaved by the addition of exogenous trypsin, our reconstruction showed continuous density for the peptide backbone throughout this region, supporting our SDS-PAGE data that indicated that the spike used in these studies had not been proteolytically processed at the S1/S2 cleavage site (Fig. 2). These data are also consistent with previous reports that PEDV S remains uncleaved during viral packaging and becomes proteolytically activated upon exposure to lysosomal proteases in the host cell (39).

The S2 subunit (Ser756 to Tyr1242) is positioned beneath the fusion-suppressive S1 capping subunit, forming extensive contacts with the internal face of the S1-CTD and subdomains 1 and 2. S2 is organized into 16 α-helices and 12 β-strands, three of which form an elongated, twisting β-sheet that resembles a fold observed previously in the fusion machinery of other class I fusion proteins. The hydrophobic fusion peptide (Ser892 to Val910) forms an α-helix that is directly C terminal to the S2′ protease cleavage site at Arg891 (Fig. S4A and C). Although there is density to show Arg891 packing tightly against S2, the “S2′ loop” spanning residues Ser878 to Gln889 could not be resolved, suggesting that this short stretch of residues does not adopt a single, rigid conformation. Overall, the S2 subunit of PEDV S strongly resembles the fusion machinery of other coronavirus spikes from diverse genera. Structural comparisons between PEDV S residues 756 to 1242 and the corresponding residues in NL63 S2, SARS-CoV S2, PDCoV S2, and infectious bronchitis virus (IBV) S2 yielded root mean square deviation (RMSD) values of 2.3 Å, 5.1 Å, 2.5 Å, and 4.1 Å, respectively (Fig. S3B) (30, 34, 38, 40). A small HR1 insertion (residues Ile1018 to Ala1031) that is present in the alphacoronaviruses and deltacoronaviruses but absent in the betacoronaviruses and gammacoronaviruses contributes to the higher level of divergence between SARS-CoV S2, IBV S2 and PEDV S2.

### D∅ architecture and PEDV PC177 S characterization.

**FIG 3:**
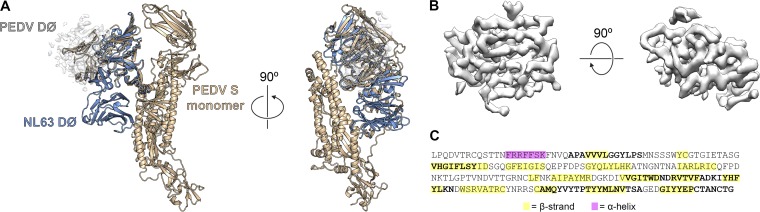
PEDV D∅ structure and architecture. (A) A single monomer of PEDV S is shown as ribbons colored tan. The density corresponding to the tan-colored D∅ is shown as a transparent surface. The NL63 NTD and D∅ are shown as blue ribbons. The NL63 NTD is shown aligned to the PEDV NTD. (B) The density for D∅ is shown as a surface colored gray. (C) The primary sequence for D∅ is shown, with residues that were included in the final PEDV S model indicated in bold. Residues that are predicted to form β-strands are colored in yellow, and residues that are predicted to form α-helices are colored in pink, based on PSIPRED secondary structure prediction.

The most dramatic difference between the structure of PEDV S and that of the previously reported alphacoronavirus NL63 S is the position of their respective D∅ domains. In NL63 S, this domain was observed tucked against S2, pointing down toward the viral membrane. However, our reconstruction of PEDV S clearly shows the corresponding density for D∅ jutting out from the apex of the trimer such that it is perpendicular to the 3-fold axis of symmetry and readily accessible to interact with its host cell attachment factor, sialic acid (Fig. 3A). The positions of D∅ in these two structures are roughly related by a 180° rotation about a horizontal axis (see Movie S1 in the supplemental material). Perhaps due to some intrinsic flexibility of this domain, a complete *de novo* model for PEDV D∅ could not be unambiguously built, and our final model for PEDV S lacks ∼100 residues in this region. However, even with the relatively poor connectivity of our map in this region, we were able to observe density that corresponds to the predicted β-sheet-rich composition of PEDV S D∅ (Fig. 3B and C).

**FIG 4:**
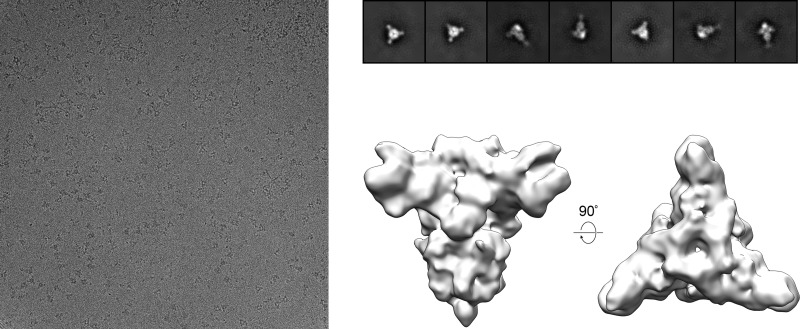
Cryo-EM screening of PEDV CV777 S in the presence of a sialic acid analog. A representative cryo-EM micrograph of PEDV CV777 S in the presence of 2 mM 3′-sialyllactose taken at ×92,000 magnification is shown on the left. 2D class averages of the spike, calculated using *cis*TEM software (49), are shown at the top right. Side and top views of the 10-Å reconstruction, calculated in *cis*TEM by imposing C3 symmetry, are shown at the bottom right.

In an attempt to determine whether the presence of sialic acid could alter the conformation of PEDV S D∅, we incubated PEDV CV777 S with an ∼750-fold molar excess of the sialic acid analog 3′-sialyllactose. Screening this complex under cryogenic conditions yielded a 10-Å reconstruction that was indistinguishable from the PEDV CV777 S apo structure (Fig. 4). These observations are consistent with the theory that host glycans such as sialic acid are likely attachment factors that do not have a dramatic influence on the conformation of the prefusion coronavirus spike (41).

**FIG 5:**
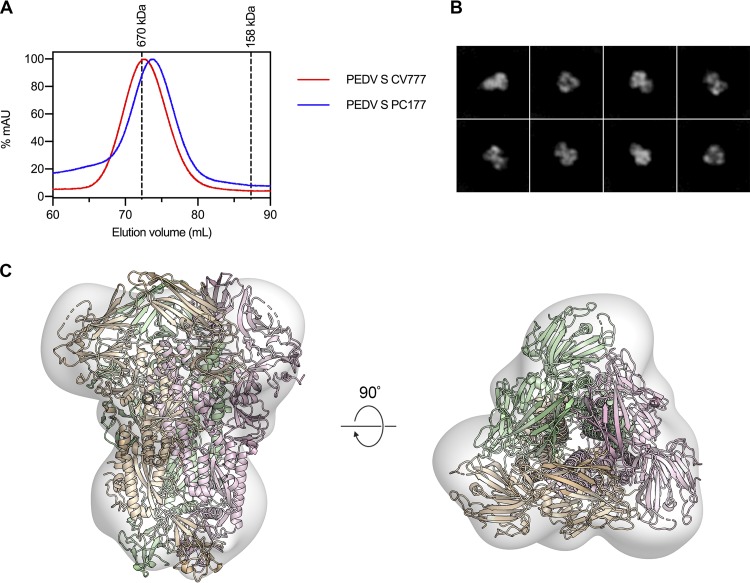
Expression and negative-stain EM analysis of PEDV PC177 S. (A) The SEC chromatograms of PEDV S CV777 and PEDV S PC177 are shown as red and blue curves, respectively. The peak elution volumes of molecular weight calibration standards are shown as dashed lines. (B) 2D class averages from negative-stain EM analysis of PEDV PC177 S. (C) Residues 228 to 1242 of PEDV CV777 S are shown as ribbons colored tan, pink, and green docked into the map of PEDV PC177 S, which is shown as a transparent volume.

Several previous studies have described the isolation of PEDV S D∅-deletion variants that have arisen spontaneously in animals as well as in cell culture (23, 24). One such strain, PEDV PC177, was recently used to inoculate piglets in a PEDV challenge model, and it was shown that not only was the primary infection with PEDV PC177 significantly attenuated compared to infection with PEDV CV777, but also the piglets inoculated with the D∅-deletion variant were significantly more susceptible to subsequent reinfection with PEDV CV777 than those that were originally inoculated with PEDV CV777 (25). In an attempt to discover a structural explanation for these observations, we expressed and purified residues 1 to 1125 of PEDV PC177 S using the same C-terminal T4 fibritin motif and affinity tags that were used to express and purify PEDV CV777 S. Excluding the D∅ deletion at the N terminus of the spike, this variant shares 100% sequence identity with PEDV CV777 S. Because of this deletion, we were able to detect a slight but measurable shift of the PEDV PC177 S SEC elution volume relative to the elution volume of PEDV CV777 S (Fig. 5A). The purified PEDV PC177 S was then observed by negative-stain EM (Fig. 5B). The final 3D reconstruction of this S variant has a resolution of ∼20 Å, which was sufficient to show that, other than the absence of D∅, the structure of PC177 S does not dramatically differ from that of CV777 S (Fig. 5C). Because the previously reported differences in susceptibility to reinfection cannot be ascribed to any large-scale conformational differences between the spikes, these data suggest that the enhanced protection against reinfection in piglets inoculated with PEDV CV777 is due to a porcine immune response against D∅.

### S1-CTD dynamics and S1 dissociation.

**FIG 6:**
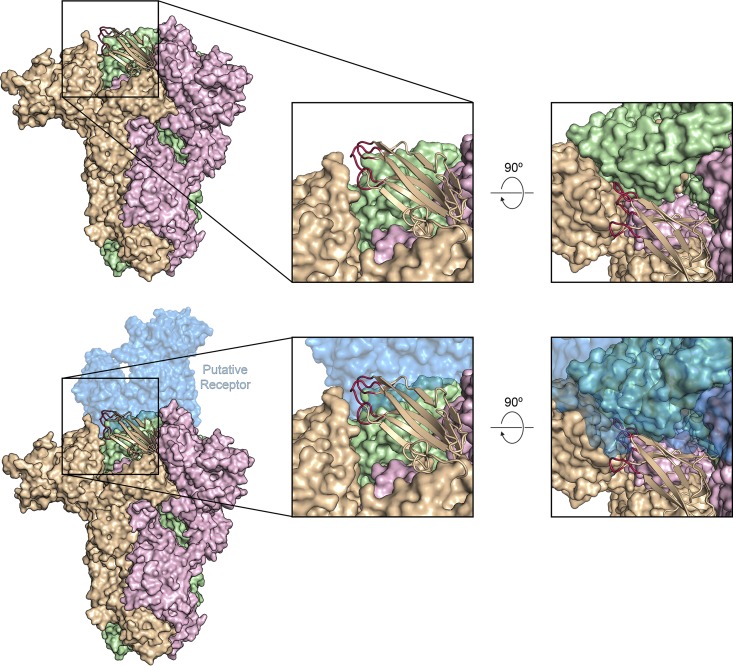
S1-CTD conformation in the intact PEDV S trimer. PEDV S is shown as a molecular surface colored tan, pink, and green. The S1-CTD of the tan protomer is shown in ribbons, and the putative receptor-binding loops are colored red. A potential receptor (porcine aminopeptidase N [PDB ID: 4F5C]) is shown as a transparent molecular surface, colored blue. This model was generated by aligning the S1-CTD of PEDV S with the PRCV RBD from the crystal structure of PRCV RBD bound to porcine aminopeptidase N (PDB ID: 4F5C).

The S1-CTDs of PEDV S adopt the same arrangement that has been described in the alphacoronaviruses and deltacoronaviruses, wherein the putative receptor-binding loops are tucked against the NTD of the same protomer (34, 38, 42). Although these loops are present at the apex of the trimer (Fig. 6, top panel; see also Fig. S5), it is unlikely that they would be able to engage a host cell protein receptor without the receptor sterically clashing with both the NTD of the same protomer and the S1-CTD of the neighboring protomer (Fig. 6, bottom panel). For the betacoronaviruses SARS-CoV and MERS-CoV, this clash is avoided when the S1-CTDs undergo a hinge-like movement that repositions the domains above the rest of S1 (31, 32). This conformational change is thought to allow the SARS-CoV and MERS-CoV S proteins to mask their determinants of receptor binding from antibodies in one conformation while making them readily accessible for host cell receptor engagement in another conformation. However, after extensive 3D classification of our PEDV CV777 data set, we were unable to observe any such conformational dynamism of the PEDV S1-CTDs in the intact PEDV S trimer. Despite this, the inaccessibility of the putative receptor-binding loops in their observed conformation suggests that there is likely to be an equivalent phenomenon in the alphacoronaviruses.

**FIG 7:**
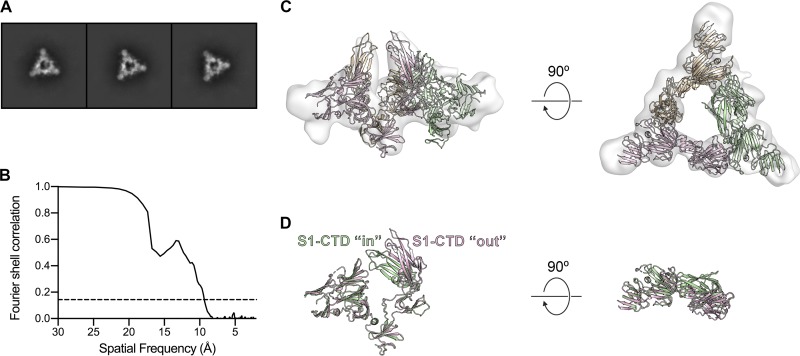
3D reconstruction of the dissociated PEDV S1 ring. (A) 2D class averages of the dissociated PEDV S1 trimer. (B) The Fourier shell correlation (FSC) curve used to calculate the resolution of the PEDV S1 ring reconstruction is shown as a solid black line and a correlation value of 0.143 is shown as a dashed line. (C) Residues 43 to 755 of PEDV S are shown as ribbons colored tan, pink, and green. The S1-CTDs of the pink and tan protomers have been repositioned to fit into the 3D reconstruction of the dissociated PEDV S1 ring, shown as a transparent surface. (D) The pink protomer and green protomer shown in panel C have been aligned to each other to highlight the conformational change of the S1-CTD.

During 2D classification of the PEDV CV777 S cryo-EM data set, roughly 10% of particles were consistently assigned into classes that resembled a trimeric S1 ring, although these classes lacked the interior density that would correspond to the S2 fusion machinery (Fig. 7A). The presence of these dissociated rings suggests that some small proportion of the purified spike was processed by endogenous trypsin-like proteases, even though SDS-PAGE analysis revealed only a single band corresponding to uncleaved S (Fig. 2). Similar dissociated S1 rings have been reported during cryo-EM analysis of MERS-CoV S (31). Despite the relatively few dissociated PEDV S1 caps that were observed, a 3D reconstruction generated from these particles resulted in a 9.4-Å map that showed evidence of the conformational rearrangement for two of the three S1-CTDs (Fig. 7B and C). These domains appear to have rotated ∼30° away from the 3-fold axis of symmetry, presumably undergoing a hinge-like motion similar to what has been observed in the betacoronaviruses (Fig. 7D). The two S1-CTDs that are observed in the “out” conformation are stacked directly on their respective SD-1s such that their receptor-binding loops are now pointed away from the NTD and are presumably readily accessible to engage with a putative host cell receptor without forming any steric clashes. Although further experimentation will be required to obtain high-resolution structures of these transient conformations, these observations provide some of the first insights into the process of alphacoronavirus receptor engagement and triggering.

## DISCUSSION

PEDV is a highly virulent alphacoronavirus that may be poised to emerge into new host populations based on its broad species tropism *in vitro*. Despite the recent determination of cryo-EM structures of coronavirus spikes, most of these efforts have been focused on the betacoronavirus genus (30–33, 43, 44), with only a single alphacoronavirus S structure reported to date (34). Given the circulation of the alphacoronaviruses HCoV-NL63 and HCoV-229E throughout the human population, it is critical that this disparity in structural information be addressed so that more-effective vaccines and therapeutics can be developed. The structure presented here of PEDV S in the prefusion conformation provides insight into the biological processes that mediate membrane fusion and has important implications for future vaccine design.

In contrast to the structure of the NL63 spike (34), our structure of PEDV S reveals an alternative D∅ conformation that would potentially make this domain more accessible to sialylated host cell surface proteins, as opposed to packing against the S2 fusion machinery. Although poor connectivity and a relatively low level of local resolution in this region of our map prevented us from reliably assigning atomic coordinates to the entirety of D∅, the map clearly reveals that this domain is located at the apex of the trimer, ∼40 Å away from the D∅ conformation in NL63 S. One possible explanation for this novel conformation is that we are merely resolving one of several alternative conformations that D∅ can sample. However, because we observed only a single, discrete conformation of D∅ in our data set, this explanation seems unlikely. It is also possible that this conformational discrepancy can be explained by the differences in the expression platforms that were used to produce the S proteins. Coronavirus S proteins are densely glycosylated, and differences in *N*-linked glycan addition and maturation in mammalian cells (used to produce PEDV S) and in insect cells (used to produce NL63 S) could lead to electrostatic differences or steric hindrances that may result in the conformational change. A third possibility is that D∅ of PEDV S genuinely adopts a stable, alternative conformation from that of the NL63 S D∅.

Our structural characterization of the PEDV PC177 spike builds upon the work of Hou et al. (25) to provide additional evidence of the immunological importance of PEDV D∅. Our ∼20-Å negative-stain EM reconstruction of PC177 S reveals that there are no large-scale conformational change differences between PC177 and CV777 S, other than the presence of D∅. Collectively, these data suggest that the differences between the host responses to infection previously observed with PC177 and CV777 were solely due to the presence or absence of D∅, suggesting that PEDV D∅ is capable of eliciting protective antibodies in pigs and that future PEDV vaccination efforts should include these crucial epitopes.

Previous structural studies of the S protein from the betacoronavirus MERS-CoV revealed dissociated, trimeric S1 rings that had not been observed previously in S proteins of any of the other three genera that make up the coronavirus family (34, 38, 40, 42). Here, for the first time in alphacoronaviruses, we report similarly dissociated PEDV S1 rings among our intact S trimers. Yuan et al. (31) were able to generate a relatively low-resolution 3D reconstruction from their dissociated MERS-CoV S1 rings, which seemed to suggest that the MERS-CoV S1-CTD was flipped into the receptor-accessible “out” conformation. The 3D reconstruction generated from dissociated PEDV S1 trimers reveals a similar phenomenon, such that two of the S1-CTDs have rotated to a receptor-accessible “out” conformation. These data provide the first evidence for conformational dynamism of the S1-CTDs in alphacoronaviruses. It is currently not known why these distinct conformational states have not been observed for intact alphacoronavirus spikes, but it is possible that without a receptor or receptor mimic such as an S1-CTD-directed antibody to trap this receptor-binding domain (RBD)-accessible conformation, the transient exposure of the S1-CTD is a very rare event (33). Nevertheless, the 2D classes and 3D reconstruction of PEDV S1 reported here provide a glimpse at how the process of alphacoronavirus spike triggering is initiated.

The presence of these dissociated spike subunits suggests a degree of protein instability that was not readily apparent based on our biochemical purification. While these S1 rings did not hinder our ability to generate a high-resolution cryo-EM map of the intact PEDV S trimer, such heterogeneity would be detrimental to attempts to vaccinate animals with a single, conformationally homogenous immunogen. It is our hope that the structure of PEDV S presented here will make it possible to rationally engineer a vaccine antigen that exhibits enhanced stability in the prefusion conformation.

## MATERIALS AND METHODS

### Protein expression and purification.

A human-codon-optimized gene fragment encoding residues 1 to 1319 of PEDV CV777 S was generously provided by Kizzmekia Corbett and Barney Graham of the Vaccine Research Center. This gene was subcloned into a pαH eukaryotic expression plasmid with a C-terminal T4 fibritin motif, an HRV3C protease cleavage site, an 8× His tag, and a Twin-Strep-tag. Further MegaPrimer subcloning was performed to remove the 194 amino acids corresponding to D∅, thereby generating a gene encoding residues 1 to 1125 of PEDV PC177 S. These plasmids were transiently transfected into FreeStyle 293-F cells (GibCo) using polyethylenimine. Transfected FreeStyle 293-F cells were treated with 5 μM kifunensine to ensure uniform high-mannose glycosylation. PEDV S CV777 and PEDV S PC177 were purified over Strep-Tactin resin (IBA Lifesciences) before being run over a Superose6 10/300 column (GE Healthcare Biosciences) in 2 mM Tris (pH 8.0), 200 mM NaCl, 0.02% NaN_3_.

### PEDV CV777 S SDS-PAGE analysis and trypsin digestion.

SEC-purified PEDV CV777 S was mixed with tosyl phenylalanyl chloromethyl ketone (TPCK)-treated trypsin from bovine pancreas (Sigma-Aldrich) at a ratio of 10:1 (wt/wt). This mixture was incubated at 37°C, and samples from various time points were collected, reduced by the addition of lithium dodecyl sulfate (LDS) loading dye containing 5% 2-mercaptoethanol, and analyzed by SDS-PAGE after being heated for 5 min at 98°C.

### Cryo-EM data collection.

CF-2/2 grids (Electron Microscopy Sciences) were plasma cleaned for 30 s in a Gatan Solarus 950 plasma cleaner with a 4:1 O_2_/H_2_ ratio. PEDV S CV777 at a concentration of 0.4 mg/ml was mixed with 2 mM Tris (pH 8.0), 200 mM NaCl, 0.02% NaN_3,_ and 0.01% Amphipol A8-35 and was deposited onto the grids before being blotted for 6 s and plunge-frozen in liquid ethane using a Vitrobot Mark IV instrument (Thermo Scientific). The frozen grid was imaged using an FEI Titan Krios transmission electron microscope (Thermo Scientific) equipped with a K2 direct electron detector (Gatan) at a nominal magnification of ×22,500, corresponding to a calibrated pixel size of 1.075 Å/pixel. A total of 3,186 movies were collected using Leginon (45). A full description of the cryo-EM data collection parameters can be found in Table 1.

**TABLE 1:** Data collection and reconstruction parameters

Parameter	Result			
Protein	PEDV S CV777	PEDV S1 CV777	PEDV S CV777 + 3′-sialyllactose	PEDV S PC177
EMDB ID	EMD-20672	EMD-20671		
Microscope	FEI Titan Krios	FEI Titan Krios	FEI Talos	FEI Talos
Voltage (kV)	300	300	200	200
Detector	Gatan K2 Summit	Gatan K2 Summit	Ceta 16M	Ceta 16M
Exposure (e^−^/Å^2^)	48	48	24	24
Defocus range (μm)	0.7–2.4	0.7–2.4	0.5–3.6	1.5–3.4
Final no. of particles	112,655	28,993	3,550	4,177
Symmetry imposed	C3	NA^*a*^ (C1)	C3	NA (C1)
Resolution (Å)	3.14	9.43	10	20

a NA, not applicable.

### Cryo-EM data processing.

Motion correction and CTF estimation were performed for PEDV S CV777 movies in *Warp* (35). Unbiased, nontemplated particle picking was also performed in *Warp* using BoxNet. BoxNet-selected particles were imported into cryoSPARC v2.4.6 (Structura Biotechnology) (36) for 2D classification, *ab initio* reconstruction, 3D classification, and nonuniform 3D refinement. The resulting 3.1-Å map was then subjected to local-resolution-guided sharpening in LocalDeblur (37). A detailed description of the cryo-EM data processing workflow can also be found in Fig. S1. Model-building was performed by fitting a small, homologous portion of the NL63 S2 fusion machinery (PDB identifier [ID] 5SZS) into the PEDV S CV777 map. The rest of the model was built manually in Coot (46) and refined in both PHENIX (47) and ISOLDE (48) (Table 2).

**TABLE 2:** Model refinement and validation statistics

Statistic	Result
PDB ID	6U7K
Composition	
Amino acids	3192
Sugars	81
RMSD bonds (Å)	0.017
RMSD angles (°)	2.29
Ramachandran	
Favored (%)	94.7
Allowed (%)	5.3
Outliers (%)	0
Rotamer outliers (%)	2.5
Clash score	3.4
MolProbity score	1.8
EM Ringer score	4.5

### Cryo-EM screening in the presence of 3′-sialyllactose.

PEDV CV777 S at a concentration of 0.4 mg/ml was incubated with 2 mM 3′-sialyllactose (Sigma-Aldrich) in 2 mM Tris (pH 8.0), 200 mM NaCl, 0.02% NaN_3_, 0.01% Amphipol A8-35 for 3 h at 4 °C. A 3-μl volume of the spike and 3′-sialyllactose mixture was deposited on a plasma-cleaned CF-2/2 grid before being blotted for 6 s and plunge-frozen in liquid ethane using a Vitrobot Mark IV instrument. The frozen grid was imaged using an FEI Talos transmission electron microscope (Thermo Scientific) equipped with a Ceta 16M detector. Micrographs were collected manually using TIA v4.14 (Thermo Scientific) at a nominal magnification of ×92,000, corresponding to a calibrated pixel size of 1.63 Å/pixel. A full description of the EM data collection parameters can be found in Table 1. CTF estimation, particle picking, 2D classification, *ab initio* 3D reconstruction, and 3D refinement were performed in *cis*TEM (49).

### Negative-stain EM data collection and processing.

PEDV S PC177 was deposited onto a plasma-cleaned CF400-Cu grid (Electron Microscopy Sciences) at a concentration of 0.035 mg/ml before being stained with methylamine tungstate (Nanoprobes). The stained grid was imaged using an FEI Talos transmission electron microscope equipped with a Ceta 16M detector. Micrographs were collected manually using TIA v4.14 at a nominal magnification of ×92,000, corresponding to a calibrated pixel size of 1.63 Å/pixel. A full description of the negative-stain EM data collection parameters can be found in Table 1. CTF estimation, particle picking, 2D classification, *ab initio* 3D reconstruction, and 3D refinement and sharpening were all performed in *cis*TEM.

### Data availability.

Cryo-EM maps have been deposited in the Electron Microscopy Data Bank (EMDB) under accession codes EMD-20671 and EMD-20672. The atomic model has been deposited in the Protein Data Bank (PDB) under accession code 6U7K.

## References

[1] Wood EN 1977 An apparently new syndrome of porcine epidemic diarrhoea. Vet Rec 100:243–244. doi:10.1136/vr.100.12.243. 888300

[2] Shibata I, Tsuda T, Mori M, Ono M, Sueyoshi M, Uruno K 2000 Isolation of porcine epidemic diarrhea virus in porcine cell cultures and experimental infection of pigs of different ages. Vet Microbiol 72:173–182. doi:10.1016/s0378-1135(99)00199-6. 10727829 PMC7117361

[3] Stevenson GW, Hoang H, Schwartz KJ, Burrough ER, Sun D, Madson D, Cooper VL, Pillatzki A, Gauger P, Schmitt BJ, Koster LG, Killian ML, Yoon KJ 2013 Emergence of porcine epidemic diarrhea virus in the United States: clinical signs, lesions, and viral genomic sequences. J Vet Diagn Invest 25:649–654. doi:10.1177/1040638713501675. 23963154

[4] Pensaert MB, de Bouck P 1978 A new coronavirus-like particle associated with diarrhea in swine. Arch Virol 58:243–247. doi:10.1007/bf01317606. 83132 PMC7086830

[5] Sellnow TL, Parker JS, Sellnow DD, Littlefield RS, Helsel EM, Getchell MC, Smith JM, Merrill SC 2017 Improving biosecurity through instructional crisis communication: lessons learned from the PEDv outbreak. J Appl Commun 101(4):2. doi:10.4148/1051-0834.1298.

[6] Alvarez J, Goede D, Morrison R, Perez A 2016 Spatial and temporal epidemiology of porcine epidemic diarrhea (PED) in the Midwest and Southeast regions of the United States. Prev Vet Med 123:155–160. doi:10.1016/j.prevetmed.2015.11.003. 26586344

[7] Sun RQ, Cai RJ, Chen YQ, Liang PS, Chen DK, Song CX 2012 Outbreak of porcine epidemic diarrhea in suckling piglets, China. Emerg Infect Dis 18:161–163. doi:10.3201/eid1801.111259. 22261231 PMC3381683

[8] Opriessnig T, Gerber PF, Shen H, de Castro A, Zhang J, Chen Q, Halbur P 2017 Evaluation of the efficacy of a commercial inactivated genogroup 2b-based porcine epidemic diarrhea virus (PEDV) vaccine and experimental live genogroup 1b exposure against 2b challenge. Vet Res 48:69. doi:10.1186/s13567-017-0472-z. 29073936 PMC5659040

[9] Kocherhans R, Bridgen A, Ackermann M, Tobler K 2001 Completion of the porcine epidemic diarrhoea coronavirus (PEDV) genome sequence. Virus Genes 23:137–144. doi:10.1023/A:1011831902219. 11724265 PMC7089135

[10] Bosch BJ, van der Zee R, de Haan CA, Rottier PJ 2003 The coronavirus spike protein is a class I virus fusion protein: structural and functional characterization of the fusion core complex. J Virol 77:8801–8811. doi:10.1128/JVI.77.16.8801-8811.2003. 12885899 PMC167208

[11] Wicht O, Li W, Willems L, Meuleman TJ, Wubbolts RW, van Kuppeveld FJ, Rottier PJ, Bosch BJ 2014 Proteolytic activation of the porcine epidemic diarrhea coronavirus spike fusion protein by trypsin in cell culture. J Virol 88:7952–7961. doi:10.1128/JVI.00297-14. 24807723 PMC4097775

[12] Li W, Luo R, He Q, van Kuppeveld FJM, Rottier PJM, Bosch BJ 2017 Aminopeptidase N is not required for porcine epidemic diarrhea virus cell entry. Virus Res 235:6–13. doi:10.1016/j.virusres.2017.03.018. 28363778 PMC7114539

[13] Milewska A, Zarebski M, Nowak P, Stozek K, Potempa J, Pyrc K 2014 Human coronavirus NL63 utilizes heparan sulfate proteoglycans for attachment to target cells. J Virol 88:13221–13230. doi:10.1128/JVI.02078-14. 25187545 PMC4249106

[14] Wang N, Shi X, Jiang L, Zhang S, Wang D, Tong P, Guo D, Fu L, Cui Y, Liu X, Arledge KC, Chen YH, Zhang L, Wang X 2013 Structure of MERS-CoV spike receptor-binding domain complexed with human receptor DPP4. Cell Res 23:986–993. doi:10.1038/cr.2013.92. 23835475 PMC3731569

[15] Wu K, Li W, Peng G, Li F 2009 Crystal structure of NL63 respiratory coronavirus receptor-binding domain complexed with its human receptor. Proc Natl Acad Sci U S A 106:19970–19974. doi:10.1073/pnas.0908837106. 19901337 PMC2785276

[16] Li F, Li W, Farzan M, Harrison SC 2005 Structure of SARS coronavirus spike receptor-binding domain complexed with receptor. Science 309:1864–1868. doi:10.1126/science.1116480. 16166518

[17] Walls AC, Tortorici MA, Snijder J, Xiong X, Bosch BJ, Rey FA, Veesler D 2017 Tectonic conformational changes of a coronavirus spike glycoprotein promote membrane fusion. Proc Natl Acad Sci U S A 114:11157–11162. doi:10.1073/pnas.1708727114. 29073020 PMC5651768

[18] Xu Y, Lou Z, Liu Y, Pang H, Tien P, Gao GF, Rao Z 2004 Crystal structure of severe acute respiratory syndrome coronavirus spike protein fusion core. J Biol Chem 279:49414–49419. doi:10.1074/jbc.M408782200. 15345712 PMC8008698

[19] Wesley RD, Woods RD, Cheung AK 1991 Genetic analysis of porcine respiratory coronavirus, an attenuated variant of transmissible gastroenteritis virus. J Virol 65:3369–3373. 1851885 10.1128/jvi.65.6.3369-3373.1991 PMC240999

[20] Li C, Li W, Lucio de Esesarte E, Guo H, van den Elzen P, Aarts E, van den Born E, Rottier PJM, Bosch BJ 26 5 2017, posting date Cell attachment domains of the porcine epidemic diarrhea virus spike protein are key targets of neutralizing antibodies. J Virol doi:10.1128/JVI.00273-17. PMC5446644 28381581

[21] Liu C, Tang J, Ma Y, Liang X, Yang Y, Peng G, Qi Q, Jiang S, Li J, Du L, Li F 2015 Receptor usage and cell entry of porcine epidemic diarrhea coronavirus. J Virol 89:6121–6125. doi:10.1128/JVI.00430-15. 25787280 PMC4442452

[22] Li W, Hulswit RJG, Widjaja I, Raj VS, McBride R, Peng W, Widagdo W, Tortorici MA, van Dieren B, Lang Y, van Lent JWM, Paulson JC, de Haan CAM, de Groot RJ, van Kuppeveld FJM, Haagmans BL, Bosch BJ 2017 Identification of sialic acid-binding function for the Middle East respiratory syndrome coronavirus spike glycoprotein. Proc Natl Acad Sci U S A 114:E8508–E8517. doi:10.1073/pnas.1712592114. 28923942 PMC5635925

[23] Diep NV, Norimine J, Sueyoshi M, Lan NT, Yamaguchi R 2017 Novel porcine epidemic diarrhea virus (PEDV) variants with large deletions in the spike (S) gene coexist with PEDV strains possessing an intact S gene in domestic pigs in Japan: a new disease situation. PLoS One 12:e0170126. doi:10.1371/journal.pone.0170126. 28095455 PMC5241010

[24] Oka T, Saif LJ, Marthaler D, Esseili MA, Meulia T, Lin CM, Vlasova AN, Jung K, Zhang Y, Wang Q 2014 Cell culture isolation and sequence analysis of genetically diverse US porcine epidemic diarrhea virus strains including a novel strain with a large deletion in the spike gene. Vet Microbiol 173:258–269. doi:10.1016/j.vetmic.2014.08.012. 25217400 PMC7126216

[25] Hou Y, Lin CM, Yokoyama M, Yount BL, Marthaler D, Douglas AL, Ghimire S, Qin Y, Baric RS, Saif LJ, Wang Q 26 6 2017, posting date Deletion of a 197-amino-acid region in the N-terminal domain of spike protein attenuates porcine epidemic diarrhea virus in piglets. J Virol doi:10.1128/JVI.00227-17. PMC5487580 28490591

[26] Wong AHM, Tomlinson ACA, Zhou D, Satkunarajah M, Chen K, Sharon C, Desforges M, Talbot PJ, Rini JM 2017 Receptor-binding loops in alphacoronavirus adaptation and evolution. Nat Commun 8:1735. doi:10.1038/s41467-017-01706-x. 29170370 PMC5701055

[27] Reguera J, Santiago C, Mudgal G, Ordono D, Enjuanes L, Casasnovas JM 2012 Structural bases of coronavirus attachment to host aminopeptidase N and its inhibition by neutralizing antibodies. PLoS Pathog 8:e1002859. doi:10.1371/journal.ppat.1002859. 22876187 PMC3410853

[28] Li BX, Ge JW, Li YJ 2007 Porcine aminopeptidase N is a functional receptor for the PEDV coronavirus. Virology 365:166–172. doi:10.1016/j.virol.2007.03.031. 17467767 PMC7103304

[29] Shirato K, Maejima M, Islam MT, Miyazaki A, Kawase M, Matsuyama S, Taguchi F 2016 Porcine aminopeptidase N is not a cellular receptor of porcine epidemic diarrhea virus, but promotes its infectivity via aminopeptidase activity. J Gen Virol 97:2528–2539. doi:10.1099/jgv.0.000563. 27449937

[30] Kirchdoerfer RN, Wang N, Pallesen J, Wrapp D, Turner HL, Cottrell CA, Corbett KS, Graham BS, McLellan JS, Ward AB 2018 Stabilized coronavirus spikes are resistant to conformational changes induced by receptor recognition or proteolysis. Sci Rep 8:15701. doi:10.1038/s41598-018-34171-7. 30356097 PMC6200764

[31] Yuan Y, Cao D, Zhang Y, Ma J, Qi J, Wang Q, Lu G, Wu Y, Yan J, Shi Y, Zhang X, Gao GF 2017 Cryo-EM structures of MERS-CoV and SARS-CoV spike glycoproteins reveal the dynamic receptor binding domains. Nat Commun 8:15092. doi:10.1038/ncomms15092. 28393837 PMC5394239

[32] Pallesen J, Wang N, Corbett KS, Wrapp D, Kirchdoerfer RN, Turner HL, Cottrell CA, Becker MM, Wang L, Shi W, Kong WP, Andres EL, Kettenbach AN, Denison MR, Chappell JD, Graham BS, Ward AB, McLellan JS 2017 Immunogenicity and structures of a rationally designed prefusion MERS-CoV spike antigen. Proc Natl Acad Sci U S A 114:E7348–E7357. doi:10.1073/pnas.1707304114. 28807998 PMC5584442

[33] Walls AC, Xiong X, Park YJ, Tortorici MA, Snijder J, Quispe J, Cameroni E, Gopal R, Dai M, Lanzavecchia A, Zambon M, Rey FA, Corti D, Veesler D 2019 Unexpected receptor functional mimicry elucidates activation of coronavirus fusion. Cell 176:1026–1039.e15. doi:10.1016/j.cell.2018.12.028. 30712865 PMC6751136

[34] Walls AC, Tortorici MA, Frenz B, Snijder J, Li W, Rey FA, DiMaio F, Bosch BJ, Veesler D 2016 Glycan shield and epitope masking of a coronavirus spike protein observed by cryo-electron microscopy. Nat Struct Mol Biol 23:899–905. doi:10.1038/nsmb.3293. 27617430 PMC5515730

[35] Tegunov D, Cramer P 2018 Real-time cryo-EM data pre-processing with *Warp*. bioRxiv doi:10.1101/338558.

[36] Punjani A, Rubinstein JL, Fleet DJ, Brubaker MA 2017 cryoSPARC: algorithms for rapid unsupervised cryo-EM structure determination. Nat Methods 14:290–296. doi:10.1038/nmeth.4169. 28165473

[37] Ramírez-Aportela E, Vilas JL, Glukhova A, Melero R, Conesa P, Martínez M, Maluenda D, Mota J, Jiménez A, Vargas J, Marabini R, Sexton PM, Carazo JM, Oscar C, Sorzano S, Cowen L 2019 Automatic local resolution-based sharpening of cryo-EM maps. Bioinformatics doi:10.1093/bioinformatics/btz671. PMC9883678 31504163

[38] Shang J, Zheng Y, Yang Y, Liu C, Geng Q, Tai W, Du L, Zhou Y, Zhang W, Li F 15 2 2018, posting date Cryo-electron microscopy structure of porcine deltacoronavirus spike protein in the prefusion state. J Virol doi:10.1128/JVI.01556-17. PMC5790952 29070693

[39] Liu C, Ma Y, Yang Y, Zheng Y, Shang J, Zhou Y, Jiang S, Du L, Li J, Li F 2016 Cell entry of porcine epidemic diarrhea coronavirus is activated by lysosomal proteases. J Biol Chem 291:24779–24786. doi:10.1074/jbc.M116.740746. 27729455 PMC5114425

[40] Shang J, Zheng Y, Yang Y, Liu C, Geng Q, Luo C, Zhang W, Li F 2018 Cryo-EM structure of infectious bronchitis coronavirus spike protein reveals structural and functional evolution of coronavirus spike proteins. PLoS Pathog 14:e1007009. doi:10.1371/journal.ppat.1007009. 29684066 PMC5933801

[41] Tortorici MA, Walls AC, Lang Y, Wang C, Li Z, Koerhuis D, Boons GJ, Bosch BJ, Rey FA, de Groot RJ, Veesler D 2019 Structural basis for human coronavirus attachment to sialic acid receptors. Nat Struct Mol Biol 26:481–489. doi:10.1038/s41594-019-0233-y. 31160783 PMC6554059

[42] Xiong X, Tortorici MA, Snijder J, Yoshioka C, Walls AC, Li W, McGuire AT, Rey FA, Bosch BJ, Veesler D 30 1 2018, posting date Glycan shield and fusion activation of a deltacoronavirus spike glycoprotein fine-tuned for enteric infections. J Virol doi:10.1128/JVI.01628-17. PMC5790929 29093093

[43] Kirchdoerfer RN, Cottrell CA, Wang N, Pallesen J, Yassine HM, Turner HL, Corbett KS, Graham BS, McLellan JS, Ward AB 2016 Pre-fusion structure of a human coronavirus spike protein. Nature 531:118–121. doi:10.1038/nature17200. 26935699 PMC4860016

[44] Walls AC, Tortorici MA, Bosch BJ, Frenz B, Rottier PJM, DiMaio F, Rey FA, Veesler D 2016 Cryo-electron microscopy structure of a coronavirus spike glycoprotein trimer. Nature 531:114–117. doi:10.1038/nature16988. 26855426 PMC5018210

[45] Carragher B, Kisseberth N, Kriegman D, Milligan RA, Potter CS, Pulokas J, Reilein A 2000 Leginon: an automated system for acquisition of images from vitreous ice specimens. J Struct Biol 132:33–45. doi:10.1006/jsbi.2000.4314. 11121305

[46] Emsley P, Cowtan K 2004 Coot: model-building tools for molecular graphics. Acta Crystallogr D Biol Crystallogr 60:2126–2132. doi:10.1107/S0907444904019158. 15572765

[47] Adams PD, Grosse-Kunstleve RW, Hung LW, Ioerger TR, McCoy AJ, Moriarty NW, Read RJ, Sacchettini JC, Sauter NK, Terwilliger TC 2002 PHENIX: building new software for automated crystallographic structure determination. Acta Crystallogr D Biol Crystallogr 58:1948–1954. doi:10.1107/s0907444902016657. 12393927

[48] Croll TI 2018 ISOLDE: a physically realistic environment for model building into low-resolution electron-density maps. Acta Crystallogr D Struct Biol 74:519–530. doi:10.1107/S2059798318002425. 29872003 PMC6096486

[49] Grant T, Rohou A, Grigorieff N 2018 cisTEM, user-friendly software for single-particle image processing. Elife 7:e3538. doi:10.7554/eLife.35383. PMC5854467 29513216

